# CCN-Based Therapeutic Peptides Modify Pancreatic Ductal Adenocarcinoma Microenvironment and Decrease Tumor Growth in Combination with Chemotherapy

**DOI:** 10.3390/cells9040952

**Published:** 2020-04-13

**Authors:** Andrea Resovi, Patrizia Borsotti, Tommaso Ceruti, Alice Passoni, Massimo Zucchetti, Alexander Berndt, Bruce L. Riser, Giulia Taraboletti, Dorina Belotti

**Affiliations:** 1 Laboratory of Tumor Microenvironment, Department of Oncology, Istituto di Ricerche Farmacologiche Mario Negri IRCCS, 24126 Bergamo, Italy; andrea.resovi@marionegri.it (A.R.); patrizia.borsotti@marionegri.it (P.B.); 2 Laboratory of Cancer Pharmacology, Department of Oncology, Istituto di Ricerche Farmacologiche Mario Negri IRCCS, 20156 Milan, Italy; tommaso.ceruti@marionegri.it (T.C.); massimo.zucchetti@marionegri.it (M.Z.); 3 Laboratory of Mass Spectrometry, Department of Environmental Health Sciences, Istituto di Ricerche Farmacologiche Mario Negri IRCCS, 20156 Milan, Italy; alice.passoni@marionegri.it; 4 Section Pathology, Institute of Legal Medicine, Jena University Hospital, D-07747 Jena, Germany; Alexander.Berndt@med.uni-jena.de; 5 BLR Bio LLC, Kenosha, WI 53140, USA; riser@blrbio.com; 6 Department of Physiology and Biophysics, and Department of Medicine Center for Cancer Cell Biology, Immunology and Infection, Rosalind Franklin University of Medicine and Science, North Chicago, IL 60064, USA

**Keywords:** PDAC, tumor microenvironment, CCN2/CCN3, matricellular proteins

## Abstract

The prominent desmoplastic stroma of pancreatic ductal adenocarcinoma (PDAC) is a determinant factor in tumor progression and a major barrier to the access of chemotherapy. The PDAC microenvironment therefore appears to be a promising therapeutic target. CCN2/CTGF is a profibrotic matricellular protein, highly present in the PDAC microenvironment and associated with disease progression. Here we have investigated the therapeutic value of the CCN2-targeting BLR100 and BLR200, two modified synthetic peptides derived from active regions of CCN3, an endogenous inhibitor of CCN2. In a murine orthotopic PDAC model, the two peptides, administered as monotherapy at low doses (approximating physiological levels of CCN3), had tumor inhibitory activity that increased with the dose. The peptides affected the tumor microenvironment, inhibiting fibrosis and vessel formation and reducing necrosis. Both peptides were active in preventing ascites formation. An increased activity was obtained in combination regimens, administering BLR100 or BLR200 with the chemotherapeutic drug gemcitabine. Pharmacokinetic analysis indicated that the improved activity of the combination was not mainly determined by the substantial increase in gemcitabine delivery to tumors, suggesting other effects on the tumor microenvironment. The beneficial remodeling of the tumor stroma supports the potential value of these CCN3-derived peptides for targeting pathways regulated by CCN2 in PDAC.

## 1. Introduction

Pancreatic ductal adenocarcinoma (PDAC) is one of the most aggressive tumor types, with limited treatment options. It is characterized by a strong desmoplastic reaction, with prominent fibrosis and extracellular matrix (ECM) deposition that, on the one hand, promote PDAC progression, influencing tumor cell proliferation, survival, and invasion and, on the other, hampers distribution and activity of chemotherapy and immunotherapy [1]. This has generated great interest for the development of agents targeting the tumor microenvironment as new therapeutic tools for PDAC treatment, particularly when used in combination with cytotoxic drugs [2]. Attempts to directly deplete PDAC associated stroma cell populations often resulted in a more aggressive tumor [3,4]. However, targeting the molecular mechanisms governing the desmoplastic reaction to affect the tumor promoting activity of the microenvironment emerged as a more promising therapeutic approach [2,5].

In this context, the unique functional properties of CCN matricellular proteins place them in an area of great interest for the design of new drugs that mimic or regulate their biological activity. The CCNs are considered to be master regulators of multiple biological functions. They are produced and secreted by a variety of cell types including many tumors [6] and are found circulating in plasma or deposited in tissues where they simultaneously bind to a variety of ECM molecules and cell receptors, translating environmental signals to the cell. The four modules that make up all but one of the six-member family (CCN5 contains only three modules) contain sites for binding different receptors. These receptors include Notch, integrins, TrkA, and heparan sulfate proteoglycans, and they are known to modulate such diverse activities as cell replication, death, adhesion, motility, and ECM production [7].

Among the CCNs, CCN2 (formerly named connective tissue growth factor, CTGF) is known to play a role in early development of a variety of tissues, but mostly in pathological conditions where it interacts with ECM molecules, growth factors, and cell receptors depending on the cellular context. CCN2 is a potent inducer of ECM production [8] and a profibrotic agent in pancreatic disease [9,10,11], renal pathology [12,13], bronchopulmonary dysplasia [14], and cardiac fibrosis [15]. Its altered expression has been associated with tumorigenesis [16].

In particular, CCN2 acts as a profibrotic molecule in PDAC [17], where it is produced by acinar cells, ductal cells, stellate cells, and fibroblasts. CCN2 plays an important role in the activation of pancreatic stellate cells, stimulating the production of ECM, proteases, and protease inhibitors and modulating the activity of growth factors and angiogenic factors. Activated stellate cells, in turn, are the major source of CCN2 during PDAC progression. We have recently demonstrated that plasma CCN2 is upregulated in PDAC patients and is a valuable potential biomarker for PDAC diagnosis and for monitoring drug response [18].

CCN2 has recently been proposed as a target for PDAC therapy. The CCN2-targeting mAb FG-3019 inhibited tumor growth and metastasis in the PANC-1 orthotopic preclinical model [19]. In genetically engineered mice *LSL-Kras^G12D^*^/*+*^*;LSL-Trp53^R172H^*^/*+*^*;Pdx-1-Cre* (KPC), FG-3019 enhanced tumor response to the chemotherapeutic drug gemcitabine [20]. FG-3019 (pamrevlumab) is currently in a Phase 3 clinical trial to evaluate its efficacy and safety as neoadjuvant treatment in combination with gemcitabine plus nab-paclitaxel in the treatment of locally advanced, unresectable pancreatic cancer.

CCN3/NOV, another member of the CCN family, acts as an endogenous inhibitor of CCN2 biological activity as well as production [21]. In particular, CCN3 inhibited CCN2 profibrotic activity in in vitro and in vivo models of renal disease, where it blocked cellular injury and prevented the conversion of mesangial cells to activated alpha-smooth muscle actin-positive fibroblast-like cells [22,23]. CCN3 also reduced skin fibrosis blocking collagen type 1 production and cell proliferation stimulated by platelet derived growth factor (PDGF) [24]. Building on the endogenous regulatory role of CCN3 on CCN2, a set of small modified peptides based on two CCN3 regions identified as responsible for this activity have been created by BLR Bio, as potential agents to treat fibrotic diseases including cancer [24].

Given the role of CCN2 in PDAC progression, this study was designed to investigate the potential value of two of these peptides, BLR100 and BLR200, for use as therapeutic agents to treat PDAC, using a PDAC model transplanted orthotopically in the pancreas of immunocompetent mice. The activity of the peptides has been evaluated in tumors formed by the FC1199 tumor cells, derived from KPC mice, the model used to first demonstrate the activity of the CCN2 inhibitor FG-3019 in PDAC. When implanted orthotopically in syngeneic mice, these cells form tumors that recapitulate the pathological features of the original tumors in genetically engineered mouse model (GEMM), as well as of human PDAC, particularly in terms of desmoplastic microenvironment. This study shows the ability of BLR100 and BLR200 to modify the PDAC microenvironment, controlling tumor growth and ascites formation, particularly in combination with chemotherapy.

## 2. Materials and Methods

### 2.1. Drugs

BLR100 and BLR200 and the control peptide (scrambled) were chemically synthesized by JPT Peptide Technologies (Berlin, Germany). BLR100 and BLR200 are based on two different 14 amino acid sequences identified from different modules in CCN3, selected for ability to interact with CCN2 and block CCN2 binding to cell receptors (Riser, B.L., Inventor, “CCN3 peptides and analogs thereof for therapeutic use.” U.S. Patent 8518,395, Issued 27 August 2013) with proprietary modifications to increase stability. The purity of the peptides used were greater than 95%, with the remaining 5% representing small unincorporated amino acid groups, as determined by HPLC analysis.

The peptides were dissolved in water at the concentration of 1 mM, stored at −80 °C, and further diluted in phosphate buffered saline immediately before use. Gemcitabine (Teva, Assago, Italy) was dissolved in saline (40 mg/mL), stored at −80 °C, and further diluted immediately before use.

### 2.2. Tumor Cells

The FC1199 pancreatic cancer cell line, derived from tumors arisen in *LSL-Kras^G12D^*^/*+*^*;LSL-Trp53^R172H^*^/*+*^*;Pdx-1-Cre* mice [25] in the C57BL/6 background, was provided by D.A. Tuveson (Cold Spring Harbor, NY, USA). Cells were cultured in Dulbecco modified Eagle’s medium (DMEM) (Gibco, ThermoFisher Scientific, Rodano, Italy) supplemented with 10% Fetal calf serum (FCS) (Euroclone, Milano, Italy) and 1% L-glutamine (Gibco). Cells were kept in culture for no more than three weeks before injection in mice and routinely tested and found free of mycoplasma infection.

### 2.3. Proliferation Assay

FC1199 were seeded into 96-well plates in DMEM supplemented with 5% FCS. At 24 h after seeding, cells were exposed to increasing concentrations of BLR100 and BLR200 alone or in combination with increasing concentrations of gemcitabine. After a 72 h incubation, cells were fixed and stained with crystal violet solution (Sigma–Aldrich, Merck Life Science, Milano, Italy). The staining was eluted with a 1:1 ethanol/0.1 M sodium citrate solution, and the absorbance at 595 nm was measured. Each condition was tested in triplicate.

### 2.4. ELISA Assays

CCN2 levels in conditioned media and cell lysate of FC1199 tumor cells were measured by ELISA (CSB-E07877m, Cusabio, Wuhan, China), according to the manufacturer’s instructions. Each sample was analyzed in duplicate.

### 2.5. In Vivo Studies

Procedures involving animals and their care were conducted in conformity with institutional guidelines that comply with national (Lgs 26/2014) and EU directives laws and policies (EEC Council Directive 2010/63) in line with guidelines for the welfare and use of animals in cancer research [26] and with the “3Rs” principle. Animal studies were approved by the Mario Negri Institute Animal Care and Use Committee and by the Italian Ministry of Health (Authorization 125/2016-PR).

Six- to eight-week old female C57BL/6 mice (Charles River Laboratories, Lecco, Italy) were maintained under specific-pathogen-free conditions, with constant temperature and humidity, and handled using aseptic procedures. FC1199 cells (5 × 10^4^) were implanted orthotopically in the pancreas as described [18]. Peptides were administered i.p. at the dose of 4.5 or 10 µg/kg, as indicated, with the schedule shown in Figure 2A and Figure 4A. Gemcitabine was administered i.v. at 40 mg/kg (see scheme in Figure 4A). Control groups received the same volume of vehicle. Mice were weighed every other day as a measure of drug toxicity. When the first mice displayed signs of distress the experiment was concluded for all experimental groups. Tumor burden was evaluated at the end of the experiment, as pancreas weight. Peritoneal ascitic fluid was collected with a syringe and the volume recorded. Animals with a collectable peritoneal fluid (≥100 µL) were considered positive for ascites. Liver of mice were analyzed for macroscopic metastases, and no metastatic foci was detected in any group.

### 2.6. Pharmacokinetics Studies

FC1199-bearing mice were treated i.p with BLR200 (10 µg/kg) from day 11 for a total of 4 treatments, every other day. On day 17, mice were treated with gemcitabine (100 mg/kg, i.v., single bolus). After 30 min and 1 h, plasma and tumors were collected for HPLC measurement of gemcitabine and metabolites. The plasma fraction added with 10 µL tetrahydrouridine (THU) 2.5 mg/mL was immediately separated by centrifugation at 3200× *g* for 15 min at 4 °C and stored at −20 °C until analysis. Tumors were immediately frozen in liquid nitrogen and stored at −20 °C until analysis. Extraction and analysis of gemcitabine (dFdc, dFdCTP, and dFdU) in tumors and plasma were carried out according to Bapiro et al. [27].

Briefly, tumors were weighted, spiked with 2′-deossicitidine as the internal standard (IS) at a final concentration of 1 ng/mg, and homogenized with ice-cold acetonitrile (ACN) 50% (v/v) containing THU 25 µg/mL in a Precellys Evolution homogenizer (Bertin Technologies S.A.S., Montigny-le-Bretonneux, France). Fifty microliters of homogenate were added with 200 µL ACN 50% (v/v), vortexed, and centrifuged at 13,200 rpm for 10 min at 4 °C. The supernatant was dried under N_2_ flux and the residue reconstituted with 100 µL of milliQ water. Concerning plasma, 25 µL were spiked with 1 µL of 10 µg/mL of IS and processed in the same way as tumors. The reconstituted tumors and plasma extracts were analyzed by injecting 2 µL in a UHPLC System connected to a LCMS-8060 (Shimadzu Scientific Instruments, Columbia, MD, USA) with a dual ion source. Chromatographic separation was achieved on an Hypercarb column, 2.1 × 100 mm, 5 µm (Thermo Fisher Scientifics, Waltham, MA, USA) fluxing mobile phase at a flow rate of 0.3 mL/min under gradient conditions. The mass spectrometer worked with an electrospray ionization source operating both in the negative and positive Multiple Reaction Monitoring mode, quantifying target ions m/z 264→59.25 for gemcitabine (positive), m/z 502→158.9 for dFdCTP (negative), m/z 263.1→220 for dFdU (negative), and m/z 228.1→112.05 for IS (positive). The lower limit of quantitation (LOQ) was 0.2 ng/mg for gemcitabine, 0.4 ng/mg for dFdU, and 0.5 ng/mg for dFdCTP in tumor samples, while it was 0.01 µg/mL for gemcitabine and 0.02 µg/mL for dFdU in plasma samples.

### 2.7. Histological and Immunohistochemical Analysis

Tumors were collected, fixed in 10% phosphate-buffered formalin, embedded in paraffin, and cut into 4 µm-thick sections. Sections were stained with Hematoxylin and Eosin (H&E) and Sirius red as described [18]. For immunohistochemical analysis of the tumor vasculature, anti-mouse CD31 antibody SZ31 (Dianova GmbH, Hamburg, Germany) followed by biotin-conjugated goat anti-rat IgG antibody (Vector Laboratories, Burlingame, CA, USA) and streptavidin-alkaline phosphatase conjugate were used [18]. CCN2 expression was evaluated using anti-CCN2 (ab6992, Abcam, Milano, Italy), followed by MACH 4 Universal HRP-Polymer (Biocare Medical, Pacheco, CA, USA) [18]. Negative controls included no-primary antibody control and the use of a specific inhibitory mouse CTGF peptide (ab7861, Abcam). Images (bright field for H&E and CD31 and polarized light for Sirius red) were acquired with Axio Imager Z2 (Zeiss, Felbach, Switzerland). Presence of fibrosis and vascular structures was analyzed using ImageJ software (https://imagej.nih.gov/) and expressed as the percentage of total tumor area. The amount of necrotic tissues in tumors was quantified by blind scoring, exploiting the difference in staining intensity between vital and necrotic tissue.

### 2.8. Statistical Analysis

Differences in proliferation and tumor growth were analyzed by one-way ANOVA followed by Tukey’s or Dunnett’s multiple comparisons test. The *p* value < 0.05 was considered significant. Statistical analysis was performed using GraphPad Prism version 8 Software (GraphPad, LaJolla, CA, USA).

## 3. Results

### 3.1. Effect of CCN-Targeting Peptides (BLR100 and BLR200) on the Orthotopic Growth of PDAC

**Figure 1:**
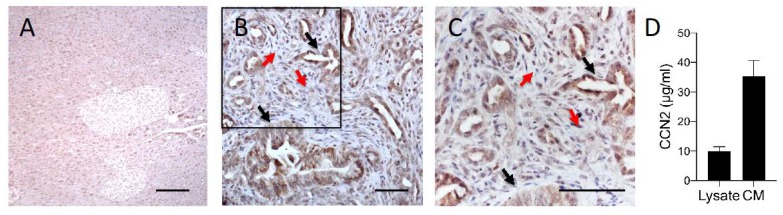
CCN2 is expressed by FC1199 tumors. CCN2 was analyzed by immunohistochemistry (IHC) in healthy murine pancreas (**A**) and FC1199 orthotopic tumors (**B**,**C**). Black arrows: tubular structures of an adenocarcinoma with cellular atypia and differences in the thickness of the tubular wall. Red arrows: stromal areas. C is a higher magnification of the boxed area in B. CCN2 was expressed by both tumor cells (black arrows) and stroma cells (red arrow), scale bars: 50 µm. (**D**) CCN2 production by FC1199 cells in vitro. CCN2 in the cell lysate and conditioned media was measured by ELISA and expressed as µg/mL.

The antitumor activity of BLR100 and BLR200 was first investigated in the murine FC1199 cells established from pancreatic tumors in KPC mice. We began by verifying that the tumor model expressed CCN2, the primary target of the peptides. Immunohistochemical analysis of FC1199 tumors grown in the pancreas of syngeneic C57BL/6 mice and characterized by the presence of significant amount of stroma, confirmed that CCN2 was expressed in vivo, both in tumor cells and stroma cells, but was barely detectable in healthy pancreas (Figure 1A–C). In agreement, FC1199 tumor cells in vitro expressed the CCN2 protein: the protein was detectable in the cell lysate and was released in relevant amounts in the conditioned media (Figure 1D). This indicated that FC1199 cells maintain the characteristics of the original KPC tumors, previously reported to produce CCN2 [18,20], and therefore represent a good model to test the therapeutic potential of CCN2-targeting agents, such as BLR100 and BLR200.

**Figure 2:**
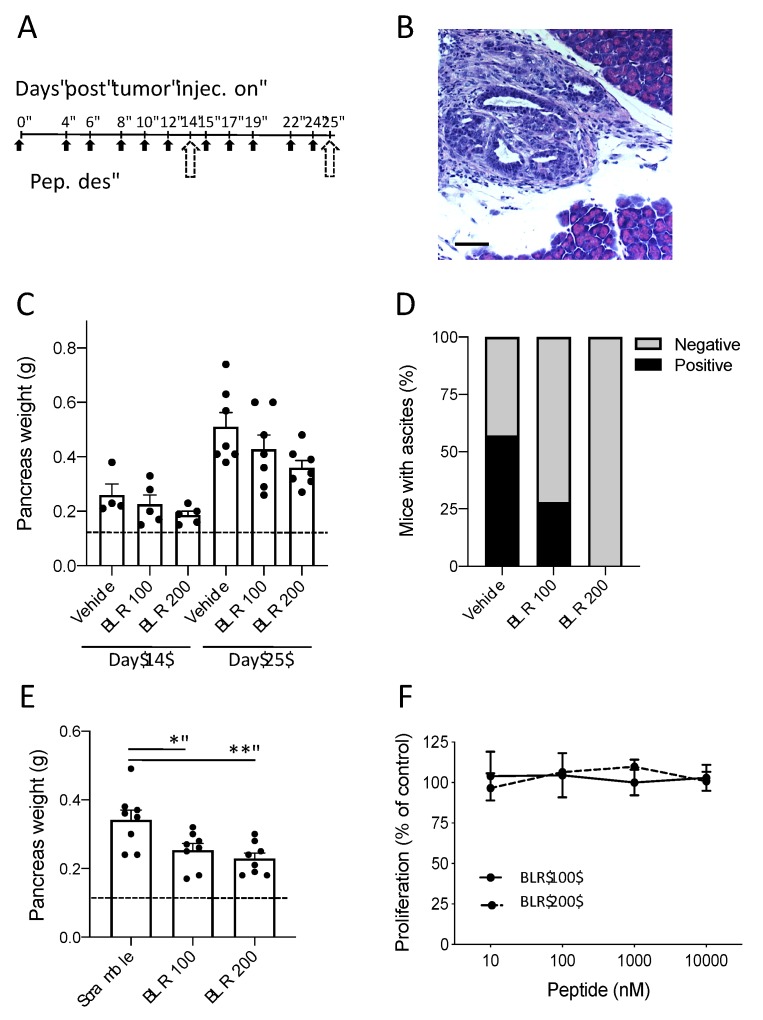
Antineoplastic activity of BLR100 or BLR200 on PDAC. (**A**) Schedule of treatment. The peptides were administered three times per week at 4.5 µg/kg, at the indicated day (black arrows). Dotted arrows indicate mice sacrifice and analysis on days 14 and 25. (**B**) Tumor in the pancreas of mice 4 days after tumor transplantation (H&E staining, scale bar 100 µm). (**C**) Tumor burden (evaluated as pancreas weight) in mice bearing FC1199 orthotopic tumors treated with vehicle, BLR100, or BLR 200 on day 14 (n = 5) or 25 (n = 7). Dotted line indicates the weight of pancreas in healthy mice. (**D**) Effect of treatments on ascites formation. Data are the percentage of mice presenting (black) or not (gray) ascites on day 25 (n = 7). (**E**) Tumor burden in mice treated with a scramble peptide, BLR100, or BLR200 (10 µg/kg) and sacrificed 25 days after tumor transplantation (n = 8). Dotted line indicates the weight of the pancreas in healthy mice. In C and E, data are mean ± SEM. One way ANOVA and Tukey’s, ** *p* < 0.005; * *p* < 0.05. (**F**) Effect of BLR100 or BLR200 on FC1199 cell proliferation in vitro. Data are the percentage of control proliferation, mean ± SEM of values from 2 independent experiments.

The activity of BLR100 and BLR200 was then tested on orthotopic, early stage FC1199 tumors, starting treatments 4 days after tumor cell injection, when tumors were histologically detectable (Figure 2A,B). The peptides were initially administered at the dose of 4.5 µg/kg to simulate a “physiological” concentration of CCN3, calculated based on the molar quantity of circulating CCN3 present in a normal mouse [23] and adjusted upward slightly to anticipate increased clearance by the kidneys of a small peptide as compared to full-length CCN3 protein. Preliminary pharmacokinetic study in healthy mice indicated that, following i.p. administration, the peptides distribute to the pancreas, where they were detected in biologically relevant concentrations (not shown). Also, our previous studies in diabetic kidney disease had shown that dosing CCN3 at 3 times per week was sufficient to block and even reverse fibrosis, so a similar schedule was selected [23]. Under these conditions, BLR100 and BLR200 were found to have a moderate, though not statistically significant, antineoplastic activity detectable as early as 10 days from the beginning of treatments (day 14) and becoming more evident at the end of the experiment (day 25, Figure 2C). Notably, the formation of ascites, a typical marker of disease progression, was prevented by both compounds. No mice treated with BLR200 had ascites in the peritoneal cavity while BLR100 treatment clearly reduced the percentage of mice with ascitic fluid (Figure 2D). Although the differences in tumors treated with BLR100 or BLR200 compared to controls did not reach statistical significance, the inhibition induced by the two peptides was reproducible and obtained with multiple preparations of the peptides. To rule out a non-specific activity of the peptides, in a subsequent experiment we used as a control a scramble peptide, consisting of the same amino acids contained in BLR200, but with a random order. Moreover, in this experiment, the dose of the peptides was increased to 10 µg/kg. Tumors treated with BLR100 and BLR200 were significantly smaller than the ones treated with the control peptide, confirming the specificity of the effect (Figure 2E).

When examined in vitro, neither BLR100 nor BLR200 affected FC1199 cell proliferation (Figure 2F), indicating that the in vivo activity was not likely due to a direct antiproliferative activity on the tumor cells and supporting the idea that the effect of two peptides on tumor growth was through a modification of the tumor microenvironment.

### 3.2. BLR100 and BLR200 Reorganize the Tumor Microenvironment

**Figure 3:**
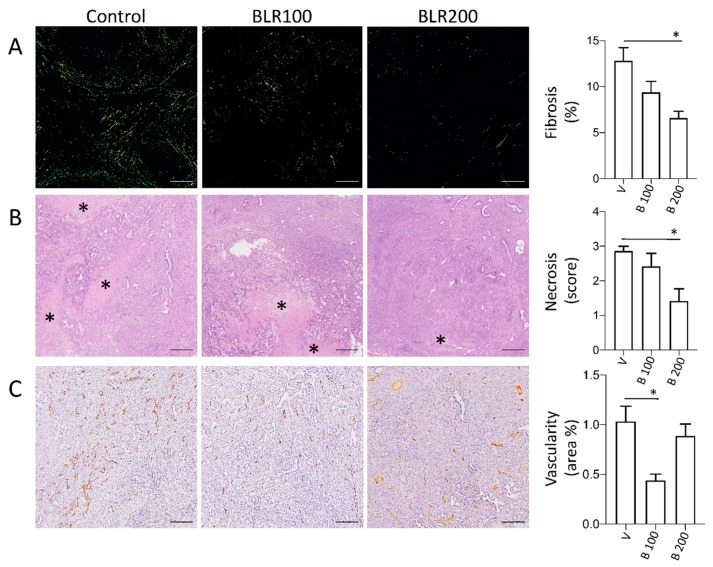
Microenvironmental changes induced by BLR100 or BLR200 on PDAC tumors. Representative images and relative quantification of (**A**) fibrosis (polarized light microscopy analysis of Sirius red stained sections; scale bars, 25 µm), (**B**) necrosis (asterisks, H&E staining; scale bars, 200 µm) and (**C**) tumor vessels (CD31 IHC; scale bars, 200 µm) of control and peptide-treated FC1199 orthotopic tumors. N ≥ 6. One way ANOVA and Dunnett’s: * *p* < 0.05.

To investigate the possible activity of the compounds on the tumor stroma, we analyzed fibrosis and neo-vascularization in tumors treated or not with BLR100 and BLR200 (4.5 µg/mL). Alterations in the tumor microenvironment were observed in treated tumors, with some differences between the two compounds. Fibrosis, a major hallmark of CCN2 activity, was reduced by treatment with both compounds. Sirius red staining of tumors (Figure 3A) showed a stromal deposition pattern of collagens (predominantly fibers appearing green in polarized light, mainly representing thin collagen fibers), which was reduced by both the peptides, although the reduction was significantly different only in BLR200 treated tumors (*p* < 0.05, Figure 3A). The fiber organization in the tumor microenvironment and the fact that this area is dominated by activated fibroblasts responsible for matrix reorganization suggests an impact of the peptides on fibroblast activity. BLR200 also relevantly reduced the areas of necrosis in the treated tumors compared with controls (*p* < 0.05, Figure 3B), whereas BLR100 was more effective in reducing tumor vascularization (*p* < 0.05, Figure 3C).

### 3.3. Antineoplastic activity of BLR100 and BLR200 in Combination with Chemotherapy

**Figure 4:**
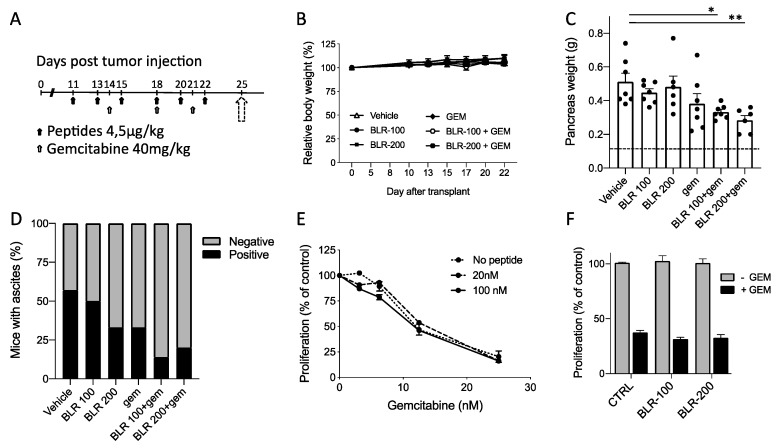
Antineoplastic activity of BLR100 or BLR200 in combination with gemcitabine on PDAC. (**A**) Schedule of treatment. Mice received the peptides (black arrows) and gemcitabine (white arrows) at the indicated times. Dotted arrow indicates mice sacrifice and tumor analysis. (**B)** Effect of the indicated treatments on mouse body weight. Data are expressed as relative body weight, the percentage of mouse weight at the beginning of treatment, mean ± SEM. (**C**) Tumor burden (pancreas weight) in mice bearing FC1199 orthotopic tumors treated with vehicles, peptide BLR100, peptide BLR200, gemcitabine (gem), or gemcitabine in combination with the peptides. Dotted line: weight of pancreas in healthy mice (n = 7, mean ± SEM, One way ANOVA and Dunnett’s * *p* < 0.05, ** *p* < 0.005). (**D**) Effect of treatments on ascites formation. Data are the percentage of mice presenting (black) or not (gray) ascites (n = 7). (**E**,**F**) BLR100 and BLR200 did not affect FC1199 cell response to gemcitabine in vitro. (**E**) Proliferation of FC1199 cell exposed to increasing concentrations of gemcitabine alone or with the peptide (20 and 100 nM). (**F**) Inhibition of FC1199 cell proliferation by gemcitabine (15 nM) in the presence of BLR100 or BLR200 (100 nM). Data are percentage of control, from one experiment representative of two.

We next investigated the antineoplastic activity of the compounds in combination with the chemotherapeutic agent gemcitabine, standard of care for PDAC. Gemcitabine was administered at the sub-optimal dose of 40 mg/kg and the peptides at the lower dose (4.5 µg/kg). The two peptides were given before and during the administration of the cytotoxic drug, to maximize the effect of microenvironmental changes on drug activity, starting treatments when tumors were palpable (corresponding to a mean pancreas weight of 0.27 g ± 0.05 g as assessed in preliminary experiments) on day 11 (Figure 4A). All treatments were well tolerated and no body weight loss was observed (Figure 4B).

Under these conditions, peptide and gemcitabine, as monotherapies, had only a marginal effect on tumor growth, as expected given the low dose of both drugs (equimolar to physiological concentrations of CCN3 for the peptides and suboptimal for gemcitabine) and the late start of peptide treatment (day 11) compared to the earlier treatment of Figure 2. However, tumors in mice treated with either BLR100 or BLR200 in combination with gemcitabine were significantly smaller than controls (*p* < 0.05 and *p* < 0.005 respectively, Figure 4C). Furthermore, the large variation in responsiveness among animals observed in the gemcitabine alone group was reduced when the combination of drugs was used. The combined treatment was also effective in reducing ascites formation, as fewer mice presented ascites in the abdominal cavity compared with mice treated with the vehicle or with the single agents (Figure 4D).

To investigate a possible direct effect of the combination on tumor cells, the effect of BLR100 at the concentrations of 20 nM or 100 nM combined with increasing doses of gemcitabine was evaluated on FC1199 cell proliferation in vitro. Figure 4E shows no difference in proliferation between cells treated with gemcitabine alone or in combination with the compound (IC50 values, in nM, were 13.5 ± 4.6, 13.2 ± 3.9, and 11.9 ± 3.7 for gemcitabine alone, and with BLR100 20 nM and 100 nM, respectively, mean and SD of two experiments). Similar findings were obtained with BLR200 (Figure 4F) indicating that the peptides do not sensitize tumor cells to gemcitabine.

### 3.4. Pharmacokinetic Studies

Since tumor vascularization, necrosis, and fibrosis are critical factors influencing drug delivery, we investigated whether the improved antineoplastic activity of the combination might be associated with a higher concentration of gemcitabine in the tumors.

**Figure 5:**
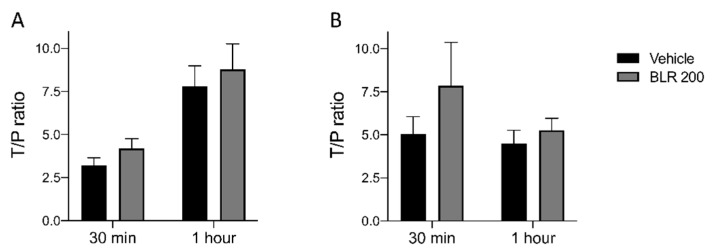
Effect of BLR200 on gemcitabine distribution. Gemcitabine (**A**) and the dFdU metabolite (**B**) were measured by HPLC–MS analysis in plasma and tumors of mice bearing FC1199 orthotopic tumors, treated or not with BLR200. Data are the ratio between tumor (T) and plasma (P) concentration, 30 min and 1 h after gemcitabine administration (mean ± SEM, n = 4).

Mice bearing FC1199 tumors were treated every other day with BLR200 (10 µg/kg) from day 11 for a total of 4 treatments. On day 17, mice were treated with gemcitabine (100 mg/kg, i.v., single bolus) and sacrificed 30 min and 1 h later. Since in vivo gemcitabine is rapidly metabolized to the active compound dFdCTP or the inactivate metabolite dFdU, we measured the concentration of both gemcitabine and its two metabolites in tumors and plasma by HPLC–MS. In BLR200-treated mice, a small increase of both native gemcitabine and the dFdU metabolite was observed, while the other metabolite, dFdCTP, was undetectable in both groups (Figure 5A,B). Although the changes induced by the peptide did not reach the level for statistical significance in these experiments, they were consistent with peptide-induced biological effect. These findings indicate that a net increase of gemcitabine concentration is not the main determinant of the improved activity of the combination, although we cannot rule out a possible effect of the peptide on the spatial distribution of gemcitabine in the tumor.

## 4. Discussion

The PDAC microenvironment is a recognized determinant of malignant progression and resistance to chemotherapy and has therefore been proposed as a target for treatments. In recent years several agents targeting the tumor microenvironment have therefore been developed, tested in preclinical PDAC models and, in some cases, also in clinical trials in combination with chemotherapy. These studies have had different outcomes depending on the nature of the agent and the clinical setting [28]. A possible reason for the variability of response might be associated with the pathways targeted by those drug candidates and the nature of the PDAC microenvironment, a complex network of interacting molecules that ultimately activate multiple pro- and antitumorigenic signals.

In this study we show that BLR100 and BLR200, two CCN2-targeting, CCN3-derived peptides, can actively modify the PDAC microenvironment and increase tumor response to chemotherapy. The study was conducted in the murine FC1199 tumor model, expressing CCN2 and growing both in vitro and in vivo, hence providing a good tool to study the activity and mechanisms of action of CCN2-targeting compounds. The lack of direct activity of BLR100 and BLR200 on tumor cell proliferation and response to gemcitabine, in vitro, appears to rule out direct cytotoxic and chemosensitizing effects of the peptides on the tumor cells, and points to a microenvironmental-mediated activity. The effect of the compounds on the microenvironment was indeed observed in FC1199 tumors in vivo, where the two peptides reduced tumor angiogenesis, fibrosis, and necrosis. Notably, the two peptides elicited a somewhat different modification of the tumors, with BLR100 appearing to be more effective in inhibiting blood vessel formation, while BLR200 appeared to be more effective at inhibiting fibrosis and necrosis. This different activity might conceivably be due to the origin of the peptides, derived from different domains of CCN3 [24], known to bind to different receptors and to carry out different functions. The finding that both peptides appear effective in PDAC opens two possible scenarios: using each peptide at different stages of disease, or combining the two peptides to simultaneously target multiple aspects of the PDAC microenvironment. Future studies will address these possibilities.

Our results are in line with the role of CCN2 in angiogenesis, vascular remodeling, and fibrosis [19]. These findings show a significant decrease in collagen deposition (predominantly fibers appearing green in polarized light, mainly representing thin collagen fibers secreted by fibroblast/mesenchymal cells), which is highly reduced by the peptides, suggesting an impact of the peptides on fibroblast activity. This is in line with described activity of the peptides that prevent procollagen synthesis by skin fibroblasts (Riser, B.L., U.S. Patent 8518,395, 2013). In preliminary studies, we found no differences in alpha smooth muscle actin (ASMA) positive cells in tumor sections of control and peptide-treated mice (not shown), possibly suggesting that the peptides might act by blocking the CCN2 effect on matrix turnover rather than fibroblast transdifferentiation into myofibroblasts. However, since our study was limited to one marker of activation, and cancer associated fibroblasts are a heterogeneous and plastic population of cells, with different levels of activation, function, and marker expression, further work will be needed to investigate the effect of the peptides on these cells.

It has been shown that CCN2 is essential for vessel maturation [29] and microvascular integrity [30]. In agreement, the CCN2-targeting monoclonal antibody FG-3019 was described as active in reducing the vasculature of the PANC-1 models [19], though not in KPC mice [20]. Two recent studies demonstrated that CCN2 inhibition (FG-3019) or deficiency (CCN2 conditional knockout) resulted in the maintenance of peritoneal function in a chlorhexidine gluconate-induced peritoneal fibrosis model by reducing angiogenesis, fibrosis, and inflammation [31,32]. Along these lines, we observed a substantial inhibition of ascitic fluid accumulation in the peritoneal cavity of mice treated with both the peptides. This finding alone may be of high importance, since in human PDAC the presence of ascites is associated with the late phases of disease progression, and is indicative of poor prognosis [33].

CCN3 is an endogenous inhibitor of CCN2, as it blocks CCN2 functions, including fibrosis, and has therefore been proposed as a model for the design of CCN2 inhibitory agents [24]. Our findings that peptides based on specific amino acid sequences of CCN3 are indeed able to inhibit fibrosis and angiogenesis confirm that CCN3 can antagonize CCN2, even in a tumor setting rich in CCN2, and supports the rationale for the development of such CCN3-based compounds for PDAC therapy. In other cell types, the peptides were able to downregulate CCN2 expression (Riser B.L., U.S. Patent 8518,395, 2013). However, preliminary data indicate that in tumors treated with peptides, CCN2 mRNA (measured by quantitative RT-PCR analysis) and protein (analyzed by IHC) were not decreased compared to controls (data not shown). This, together with the observed ability to reduce fibrosis and angiogenesis (known to be induced by CCN2) indicate that the peptides may act by inhibiting CCN2 activity rather than expression.

Both BLR peptides potentiated the therapeutic effect of gemcitabine. This indicates an additive/synergistic effect of the drugs. The finding that no synergism/additivity was observed in vitro, as the peptides did not increase tumor cell response to gemcitabine, points to a two-compartment activity of the combination, targeting tumor cell proliferation (gemcitabine) and the microenvironment (BLR drugs).

The observed reduction in fibrosis, a major obstacle to drug delivery in tumors [34], prompted us to investigate whether BLR200 might alter gemcitabine distribution. Pharmacokinetic studies indicated a small increase in gemcitabine and dFdU concentration in tumors treated with BLR200. A study using a different CCN2 inhibitory agent, FG-3019, found that the antibody was able to promote KPC tumor response to gemcitabine, without improving drug distribution. However, differently from our findings, CCN2 targeting with FG-3019 did not cause alterations in the tumor microenvironment, suggesting possible differences in the mechanisms of CCN2 inhibition between the antibody and the BLR peptides. In addition to differences in physical properties, FG-3019 would be expected to be highly specific for CCN2, whereas the CCN3 peptides might affect CCN2 and other CCN3-interacting CCNs. Preliminary studies by one of us (BLR) have shown that BLR200 treatment is able to counteract the increase in CCN1 (formerly named Cyr61) in a model of inflammatory skin fibrosis (unpublished data), in agreement with the known role of CCN3 in downregulating CCN1 [35]. Interestingly CCN1 is detected in the early precursor lesions, and intensifies with disease progression in PDAC [36] and has supporting activity in pancreatic cancer growth, invasiveness, and drug resistance [37,38]. Therefore BLR peptides might simultaneously target multiple CCN family members with protumorigenic functions in PDAC.

An additional factor to be considered is the decrease in tumor necrosis following peptide treatment. Necrosis is a major obstacle to drug delivery to tumors. In line with the findings by Cesca et al. [39] we can hypothesize that the presence of large vital areas in BLR200-treated tumors facilitates a more homogeneous distribution of the cytotoxic drug, thus promoting a greater antitumor activity of gemcitabine even in the absence of a marked increase in total drug concentration. Further studies with technologies enabling the spatial analysis of drug distribution in tumors, such as matrix-assisted laser desorption ionization-mass spectrometry imaging, are warranted to address this hypothesis.

In conclusion, this study has shown the therapeutic potential of two CCN3-based peptides in combination with gemcitabine in PDAC. In comparison to other recently developed drugs, the approach proposed here could have a great advantage since CCNs are master regulators of multiple critical disease pathways. Indeed, we observed in this study that BLR100 and BLR200 were able to affect fibrosis deposition, angiogenesis, necrosis, and ascites formation. The different ability of the two peptides to affect distinct aspects of tumor progression suggests that they may be used either in combination or at different stages of the disease. Moreover, the increased activity of the combination regimens of the peptides with gemcitabine opens new perspectives for the use of the peptides to augment the limited efficacy of current chemotherapy and perhaps also immunotherapy in PDAC. Since the increased activity of the combination treatment was obtained with suboptimal doses of gemcitabine, the use of low, “physiological” doses of these peptides might also have interesting clinical implications for the possibility of reducing dosing of the highly toxic gemcitabine while achieving a good therapeutic response. Developing new therapeutic approaches and targeting different features of tumor microenvironment, combined with the standard of care therapy is critical to increase PDAC patient survival.
